# Aging Clocks

**DOI:** 10.1093/geroni/igaa057.2982

**Published:** 2020-12-16

**Authors:** Morgan Levine

**Affiliations:** Yale University School of Medicine, New Haven, Connecticut, United States

## Abstract

While chronological age is arguably the strongest risk factor for death, disease, and disability, same-aged individuals remain heterogeneous in their susceptibilities to these various outcomes. One explanation is that chronological age is an imperfect proxy of the degree of biological aging an individual has undergone. Thus, defining measurable estimates of ‘biological age’ (in contrast to chronological age) has become a major initiative in Geroscience research. Such biomarkers of aging, or ‘aging clocks’ will 1) help identify underlying mechanisms of aging, 2) enable identification of at-risk individuals prior to disease onset, and 3) provide outcomes to assess efficacy of interventions. In this session, I will describe the various aging clocks, how they were developed, and what they track. I will also describe how aging clocks can facilitate research both within and outside of the biological sciences.

